# Discovery of new chromosomal markers through repeatome analysis of *Caryophyllaeus laticeps* (Caryophyllidea)

**DOI:** 10.1007/s00436-025-08530-z

**Published:** 2025-07-18

**Authors:** Anna Marková, Martina Orosová

**Affiliations:** Institute of Parasitology, Slovak Academy of Sciences, Hlinkova 3, 040 01 Košice, Slovakia

**Keywords:** Fluorescence *in situ* hybridization, U1 snDNA, rRNA, RepeatExplorer2, Chromosomal aberrations

## Abstract

Genomic repetitive elements are essential structural and functional components of most eukaryotes and have attracted the attention of researchers in recent years. Despite the well-documented features of the karyotype of the tapeworm species *Caryophyllaeus laticeps*, knowledge about the content and distribution of repetitive elements on its chromosomes is limited. To gain a more comprehensive understanding of the composition of the repetitive DNA, we used a combination of bioinformatic, cytogenetic and molecular techniques to characterise the repetitive DNA content of this species. RepeatExplorer2 analysis revealed that transposable elements are predominantly present in the *C. laticeps* genome. The most suitable repeat families were selected for fluorescence mapping on the chromosomes, and most of them showed a scattered hybridisation pattern. In this study, we identified three new chromosomal markers: small nuclear U1 DNA (snDNA), the 5S ribosomal DNA (5S rDNA) and a satellite DNA named ClatSat14-167, each of which hybridises to a single locus at different chromosomal locations, allowing accurate identification of three pairs of chromosomes. These markers could be very valuable tools for future evolutionary studies and greatly enrich the limited cytogenetic data for tapeworms. In addition, an increased frequency of chromosomal aberrations was detected, reaffirming previous findings on the possible adverse effects of environmental pollution on the chromosomes of the species studied.

**Supplementary Information:**

The online version contains supplementary material available at 10.1007/s00436-025-08530-z.

## Introduction

The entire collection of repetitive DNA sequences within a genome is referred to as the repeatome. These sequences are repeated hundreds or thousands of times and make up more than 50% of the total DNA content in most species. The repeatome includes various types of repetitive sequences, with transposable elements (TEs) and satellite DNAs (satDNAs) predominating (Lower et al. 2018; Louzada et al. 2020). Satellite DNA consists of long arrays of tandemly repeated sequences and is typically the major component of heterochromatin found in some vital chromosomal structures such as telomeres and centromeres (Plohl et al. 2012), although the existence of short repeats scattered throughout euchromatin has also been demonstrated (Rico-Porras et al. 2024). Transposable elements are DNA sequences that are able to move within the genome. While TEs are generally dispersed throughout the genome, some of them may exhibit local enrichment or depletion in specific chromosomal regions such as telomeres and centromeres (Biscotti et al. 2015; Wells and Feschotte 2020).

In recent years, there has been a surge in repeatome studies across various plant and animal species (e.g. Baeza and González 2021; Muravenko et al. 2022; Hejníčková et al. 2023; Samatadze et al. 2023; Toma et al. 2024; Marková et al. 2025). Initially considered “junk DNA”, repetitive DNA is now recognized for its important roles in regulating gene expression, maintaining chromosomal integrity and genome stability (Bennetzen and Wang 2014; Mehrotra and Goyal 2014; Garrido-Ramos 2015). Compared to coding sequences, repetitive DNAs evolve more rapidly, and their variable abundance, high sequence variation and distinct chromosomal distribution contribute to genomic divergence between species (Kuo et al. 2021). Despite the growing interest in repeatome research, knowledge of repetitive DNA in tapeworms remains limited. Most current knowledge comes from genomic studies that only partially address the proportion and diversity of repetitive elements in specific species (Kamenetzky et al. 2021). To address this gap, we performed the first repeatome analysis of the tapeworm *Caryophyllaeus laticeps*, an intestinal parasite of a variety of cyprinids. This species belongs to the order Caryophyllidea, which has recently undergone a phylogenetic reconstruction that has fundamentally changed the taxonomic position of some species (Scholz et al. 2021). Chromosomal analyses, in particular the chromosomal localisation of major ribosomal genes, provided more informative insights into evolutionary pathways. So far, cytogenetic studies have been performed on 25 species of this order, with molecular cytogenetic techniques applied to only seven species within the family Caryophyllaeidae (Orosová et al. 2010a, b; Kráľová-Hromadová et al. 2010; Orosová and Oros 2012; Bombarová and Špakulová 2015; Orosová et al. 2019, 2022). The available data suggest that a single rDNA locus represents the ancestral state in the order Caryophyllidea, while karyotypes with multiple loci are considered derived—a hypothesis proposed by Orosová et al. (2022). Overall, these cytogenetic data have expanded our understanding of evolutionary pathways within specific families and supported the phylogenetic reconstruction of the order Caryophyllidea (Scholz et al. 2021). This underscores the importance of integrating classical and molecular cytogenetics to improve the accuracy and reliability of such analyses.

The karyotype of *C. laticeps* has been well documented in three geographically distant populations, one from the Volga River in Russia (Petkevičiūtė and Kuperman 1992) and two from Slovakian waters—the Tisa River (Bombarová and Špakulová 2015) and the Zemplínska Šírava Reservoir (Orosová et al. 2022). In all three populations, the basic cytogenetic characteristics (number, morphology and size of chromosomes) were consistent. The karyotype consists of 10 pairs of chromosomes, exclusively bi-armed metacentric chromosomes (2n = 20; n = 10 m). In terms of internal chromosome structure and fluorescence mapping, previous molecular cytogenetic analyses have investigated the distribution of AT- and GC-rich heterochromatin and the localisation of major ribosomal genes on the chromosomes. Physical mapping revealed a single nucleolus-organiser region (NOR) locus on a small metacentric chromosome pair No. 7 and only low heterochromatin content (Orosová et al. 2022).

RepeatExplorer2 (RE) is a bioinformatics tool that enables the identification and characterisation of repetitive elements using low-coverage Illumina sequencing data, without the need for genome assembly or a reference genome (Novák et al. 2017, 2020). These advantages make RepeatExplorer2 an efficient, rapid, and cost-effective tool for repeatome analysis. In this study, we applied, for the first time, an integrated approach combining bioinformatics, molecular techniques, and cytogenetics to characterise the repetitive DNA content in the genome of a parasitic flatworm. Specifically, our aim was to identify repetitive DNA elements in the genome of *C. laticeps* and to develop novel chromosomal markers. These markers will contribute to a more precise karyotype characterisation and chromosome identification, and ultimately support comparative cytogenetic studies and evolutionary analyses as soon as data from additional tapeworm species become available.

## Material and methods

### Parasite collection and chromosome slide preparation

Specimens of *C. laticeps* for karyological analysis were obtained from common breams (*Abramis brama*) caught in the Zemplínska Šírava water reservoir (ZŠ; 48°47′09.0″N 21°57′20.5″E), a site heavily polluted with polychlorinated biphenyls (PCB). Collections were conducted during the spring of 2023 under the permit No. 48/2023 issued by the Ministry of Environment of the Slovak Republic. Fish hosts were either dissected on site or transported to the laboratory for processing. The fish were handled in accordance with the relevant guidelines and regulations (Decree of the Ministry of the Slovak Republic No. 381/2018 Coll. and Act No. 216/2018 Coll. on Fisheries) and we confirm that all methods are reported in accordance with the ARRIVE guidelines (Percie du Sert et al. 2020).

In total, 79 adult specimens of *C. laticeps* were collected from 11 out of 20 breams. The collected parasites were rinsed several times in 0.9% saline solution immediately after isolation from the host intestine. Morphological identification of the tapeworms was performed microscopically, confirming all specimens as morphotype I, as defined by Hanzelová et al. (2015). Body parts of some individuals were fixed in absolute ethanol for DNA extraction.

For cytogenetic analyses, whole living specimens were placed in 0.025% colchicine solution for one hour at room temperature (RT), followed by hypotonic treatment (0.075 M KCl) for three hours. During this incubation, the body was carefully disrupted with insulin syringes. Lastly, they were fixed in freshly prepared modified Carnoy´s fixative (methanol/acetic acid = 3:1 v/v) twice for 15 min, and stored at −20 °C. Chromosome spreads were prepared using the"hot plate"technique (Orosová and Špakulová 2018), then dehydrated in an ethanol series (70, 80 and 100%, 1 min each), dried at RT, and stored at −20 °C until further use.

### Genome sequencing, satellitome characterization and data processing

High molecular weight genomic DNA (HMW gDNA) was isolated using the cetyltrimethylammonium bromide (CTAB) method (Winnepenninckx et al. 1993). The quality and concentration of DNA was assessed using the Qubit 4.0 fluorometer (Invitrogen, Carlsbad, USA) and the Nanodrop 2000 spectrophotometer (Thermo Fisher Scientific, Waltham, MA, USA). Two samples of optimal quality were selected for low-coverage genome sequencing. A total of 20 µg of gDNA was sequenced on a HiSeq 4000 platform at Novogene (HK) Co., Ltd. (Hong Kong, China), yielding approximately 1.4 Gb of 150 bp paired-end reads with low coverage.

Sequenced reads were processed beginning with a quality check using FastQC (v0.10.1) (Andrews 2010). The data were then trimmed using Trimmomatic (v0.32) (Bolger et al. 2014), removing Illumina adapters and low-quality regions of the reads, resulting in a final read length of 135 bp. Post-trimming, quality was re-evaluated with FastQC. Repeatome analysis was performed using the RepeatExplorer2/TAREAN pipeline (Novák et al. 2017, 2020) within the Galaxy platform (https://repeatexplorer-elixir.cerit-sc.cz). A sample of 500,000 paired-end reads was analysed using the default settings and the Metazoa (v3.0) database for automatic annotation.

### Preparation of fluorescent labelled probes for fluorescent mapping

Based on the RE results, we selected 17 repeat sequences most suitable for physical mapping on chromosomes based on their genome proportions, monomer lengths, and the number of aligned reads (minimum 200) (Supplementary Table S1). The naming of these repetitive sequences followed a standardised system: the repeat name begins with the species abbreviation (Clat), followed by the either “Sat” or “TE” according to the RE annotation, a number indicating the order of decreasing repeat abundance in the genome, and the length of the consensus monomer (Ruiz-Ruano et al. 2016). Primers for these repeats were designed using the Geneious Prime software (v2021.1.1) and used for PCR amplification to generate probes for in situ hybridisation experiments (Table 1). Of the 17 selected repeats, eight repeats were successfully amplified, labelled and mapped on chromosomes of *C. laticeps*. PCR amplification for these eight repeats was performed using *C. laticeps* HMW gDNA under the following conditions: initial denaturation at 95 °C for 3 min, followed by 35 cycles of 95 °C for 30 s, from 56 to 60 °C for 30 s (specific to each repeat, see Table 1), and 72 °C for 90 s; with a final extension at 72 °C for 5 min. PCR products of the correct size were purified from a 1.5% agarose gel in 1 × TAE buffer using the Wizard SV Gel and PCR Clean-Up System (Promega). The purified products were then labelled with biotin-16-dUTP or digoxigenin-11-dUTP (both Roche Diagnostics, Mannheim, Germany) via PCR. Nine repeats failed in PCR or fluorescence in situ hybridization (FISH) (see details in Supplementary Table S1).

**Table 1:** Primer nomenclature, forward and reverse sequences, amplification temperature and size of the amplified DNA repeat

Cluster	Set of primers	Annealing (temperature, time)	Product size (bp)
ClatTE01-4757	For 5′-TGCGATAGTCTCCACAGGGA-3′ Rev 5′-CATTGGACAAGGCCCTGCTA-3′	59 °C, 30 s	1238
ClatSat02-1340	For 5′-GGAGGAGGCATGACAGATCG-3′ Rev 5′-CGAAATTCGCGTACGCCATT-3′	56 °C, 30 s	1271
ClatTE02-4765	For 5′-CCAACTCAAGTGCAAGCAGT-3′ Rev 5′-AATGGTGGATGTCGTTGCTG-3′	59 °C, 30 s	227
ClatSat05-467	For 5′-CTTGAGCTATCCTGGCACGG-3′ Rev 5′-CCGGTGCGAATCCATGTAGT-3′	60 °C, 30 s	321
ClatTE03-4582	For 5′-AATCTGGCGTCTGGAACTGA-3′ Rev 5′-TCTGTTGGCATTTCACGTCC-3′	59 °C, 30 s	245
ClatSat09-328	For 5′-GCTGGATTCTGGTCACTGCT-3′ Rev 5′-GTTGGGGAACAACGAGCAAC-3′	60 °C, 30 s	200
ClatSat13-761 (U1)	For 5′-CTCACTGCAGTCTGTTCGGT-3′ Rev5′-CTATACACCCGAGCGAACCC-3′	60 °C, 30 s	546
ClatSat14-167	For 5′-AGCTGGGTGAATGGATGGAT-3′ Rev 5′-GTGGACACTATTGATTCAAGCAA-3′	60 °C, 30 s	125

New primers were designed for the 5S rDNA based on the 5S rDNA sequence of *Schistocephalus solidus* available in the 5S rRNA database (http://combio.pl/rrna/): Cest-5S-F (5′-CAGCGACCAAACCACGTAGA-3′) and Cest-5S-R (5′-TCAGCAACACCCGGTATTCC-3′). The 5S rDNA fragment of *C. laticeps* was amplified using this new set of primers by PCR under the following conditions: 95 °C for 3 min, 35 cycles of 95 °C for 30 s, 60 °C for 30 s, and 72 °C for 1 min; with a final extension at 72 °C for 5 min. The resulting PCR product (~ 120 bp) was purified from a 1.5% agarose gel using the Wizard SV Gel and PCR Clean-Up System (Promega) and subsequently sequenced (SEQme, Dobříš, Czech Republic). The 5S rDNA FISH probe was labelled by PCR with digoxigenin-11-dUTP (Roche Diagnostics, Mannheim, Germany).

The labelled probes were used in single- or two-colour FISH following the standard protocol for cestodes (Orosová and Špakulová 2018). Slides were pre-treated with freshly prepared pepsin solution for 5 min at 37 °C in Coplin jar and then washed twice in 2 × SSC for 5 min each. Slides were digested with 100 µg mL^−1^ RNase A in 2 × SSC for one hour at 37 °C, washed twice in 2 × SSC for 5 min, with subsequent incubation in 5 × Denhard´s solution for 30 min at 37 °C. Chromosomal DNA on the slides was denatured at 68 °C for 3 min and 30 s, and probes at 90 °C for 5 min. The hybridisation solution for one slide (10 µl; 50% deionized formamide, 10% dextran sulfate in 2 × SSC) contained approximately 50 ng of labelled probes and 25 µg of sonicated salmon sperm DNA (Sigma-Aldrich). Hybridization was performed overnight at 37 °C and followed by stringent washes 2 × SSC (5 × 2 min, 46 °C), 0.1 × SSC (3 × 5 min, 62 °C) and 4 × SSC containing 0.1% Tween 20 (3 × 3 min, 37 °C). Detection and amplification of hybridization signals was performed using either a three-step detection, Cy3-conjugated streptavidin (Jackson ImmunoRes. Labs. Inc., West Grove, PA, USA) amplified with biotinylated anti-streptavidin (Vector Labs. Inc., Burlingame, CA, USA) and re-detected with Cy3-conjugated streptavidin, or a two-step detection using Alexa Fluor® 488-Monoclonal Mouse Anti-Digoxin Antibody, followed by amplification with Alexa Fluor® 488-F(ab')2 Goat Anti-Mouse IgG (H + L) (min X) secondary Antibody (both Jackson ImmunoRes. Labs. Inc., West Grove, PA, USA). Chromosomes were counterstained with DAPI in DABCO Antifade (Invitrogen, Carlsbad, CA, USA) and the slides were sealed with nail polish and stored in the dark at 4 °C prior to examination. Images were captured using a LEICA DM 4000 B microscope with a DFC 450 C digital camera. Image processing, including pseudo-colouring, merging and adjustments, was performed using Adobe Photoshop v7.0.

## Results

Clustering analysis identified 351,268 reads grouped into 9487 clusters, of which 335 clusters had genome proportions exceeding 0.01%. Automatic annotation by RE revealed that transposable elements were the predominant repetitive sequences in the *C. laticeps* genome. Class I elements, particularly Long Interspersed Nuclear Elements (LINEs), were the most abundant, comprising 98 clusters. Class I Long Terminal Repeat (LTR) elements, including Ty-3/Gypsy and Penelope, accounted for 18 and 32 clusters, respectively. In addition, 21 clusters were identified as satellite DNA, while a large portion of repetitive elements families (161 clusters) remained “unclassified” according to the RE annotation.

Seventeen selected repeats were analysed using the BLAST tool and the free version of the GIRI/RepBase database (Supplementary Table 1). As there is no dedicated database for invertebrates, Platyhelminthes, or any relatively closely related taxa in the free version of GIRI/RepBase, annotations were performed using the general Eukaryota dataset. For most of analysed repeats, no significant similarity to previously described repetitive elements was detected. However, four repeats (ClatTE01-4757, ClatTE02-4765, ClatTE03-4582, ClatSat08-4433) showed moderate similarity (~ 65%) but significantly high scores (~ 1000) and long alignment lengths (> 2000 bp) with Gypsy elements, suggesting a possible affiliation with this transposable element family. Moreover, one repeat, ClatSat13-761, showed moderate similarity (72%) to U1 snRNA of sea urchin (*Lytechinus variegatus*, accession number X01749) in RepBase. The same repeat was annotated by BLAST as U1 snRNA of *Echinococcus multilocularis* (accession number M73768.1) with notably high similarity of 91.57%. Newly designed primers successfully amplified ClatSat13-761, generating a product of approximately 500 bp, and Sanger sequencing yielded a 546 bp sequence. This sequence showed a high degree of similarity (89.8%) with a 135 bp segment of the U1 snRNA of *E. multilocularis*. The sequence of the U1 snDNA of *C. laticeps* was deposited in GenBank under the accession number PV288798.

The 5S rDNA repeat was found to be 130 bp long and showed high similarity to 5S rDNAs from various organisms, including 100% identity with the 5S rDNA of *Alexandromys fortis* (accession number XR007590382). The 5S rDNA sequence of *C. laticeps* has been deposited in GenBank under accession number PV396848.1.

**Fig. 1:**
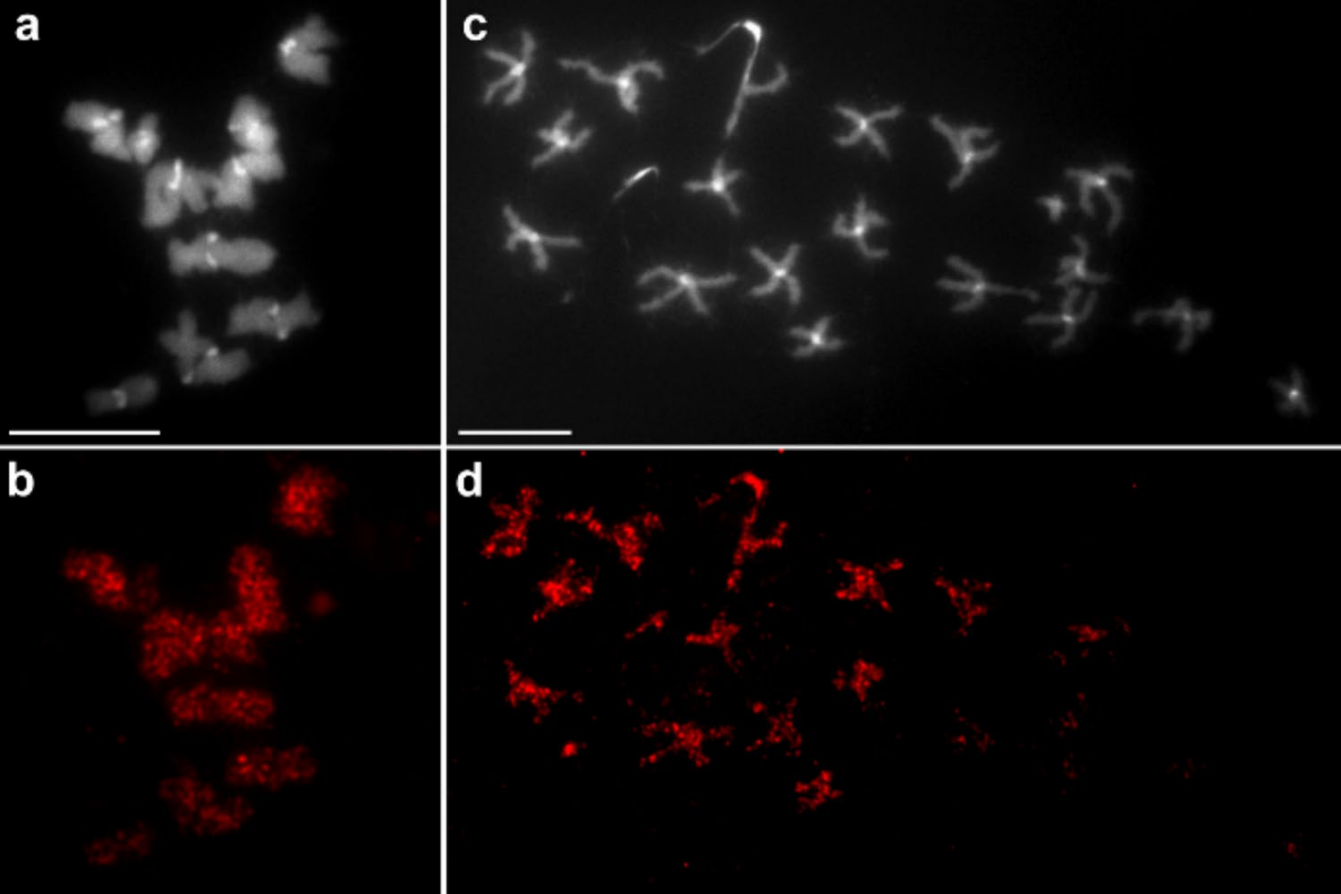
FISH mapping of repetitive elements (**a**, **b**) ClatTE01-4757 and (**c**, **d**) ClatSat05-467 on chromosomes of *Caryophyllaeus laticeps*. (**a**) DAPI-stained diplotene chromosomes with highlighted centromeres, (**b**) hybridization signals of the ClatTE01-4757 repeat, (**c**) DAPI-stained metaphase chromosomes with highlighted centromeres, (**d**) hybridization signals of repetitive element ClatSat05-467. FISH analysis revealed a dispersed distribution pattern for both repeats, with signal density decreasing with chromosome length. Scale bar = 10 μm

**Fig. 2:**
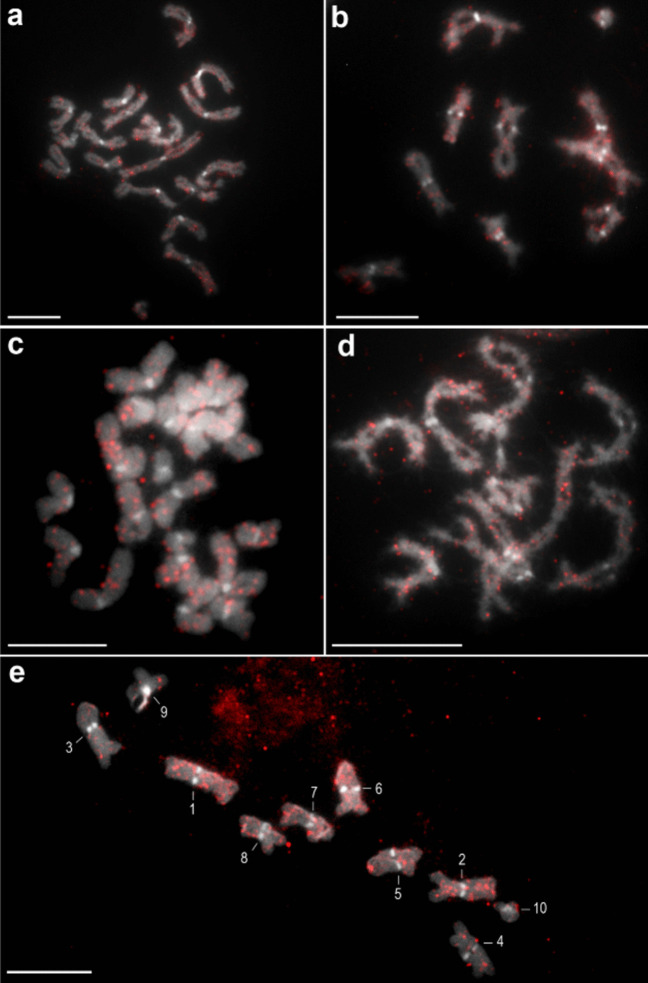
FISH with (**a**, **b**) ClatTE02-4765, (**c**, **d**) ClatTE03-4582 and (**e**) ClatSat09-328 as probes on chromosomes of *Caryophyllaeus laticeps*. (**a**) ClatTE02-4765 hybridization signals on metaphase and (**b**) diplotene nucleus, (**c**) ClatTE03-4582 hybridization signals on metaphase and (**d**) diplotene chromosomes, (**e**) ClatSat09-328 hybridization signals on diplotene nucleus, with prominent signal enrichment observed on chromosome pairs No. 1, 2, 5, 6, 7, and 8. Chromosomes were counterstained with DAPI. Scale bar = 10 μm

**Fig. 3:**
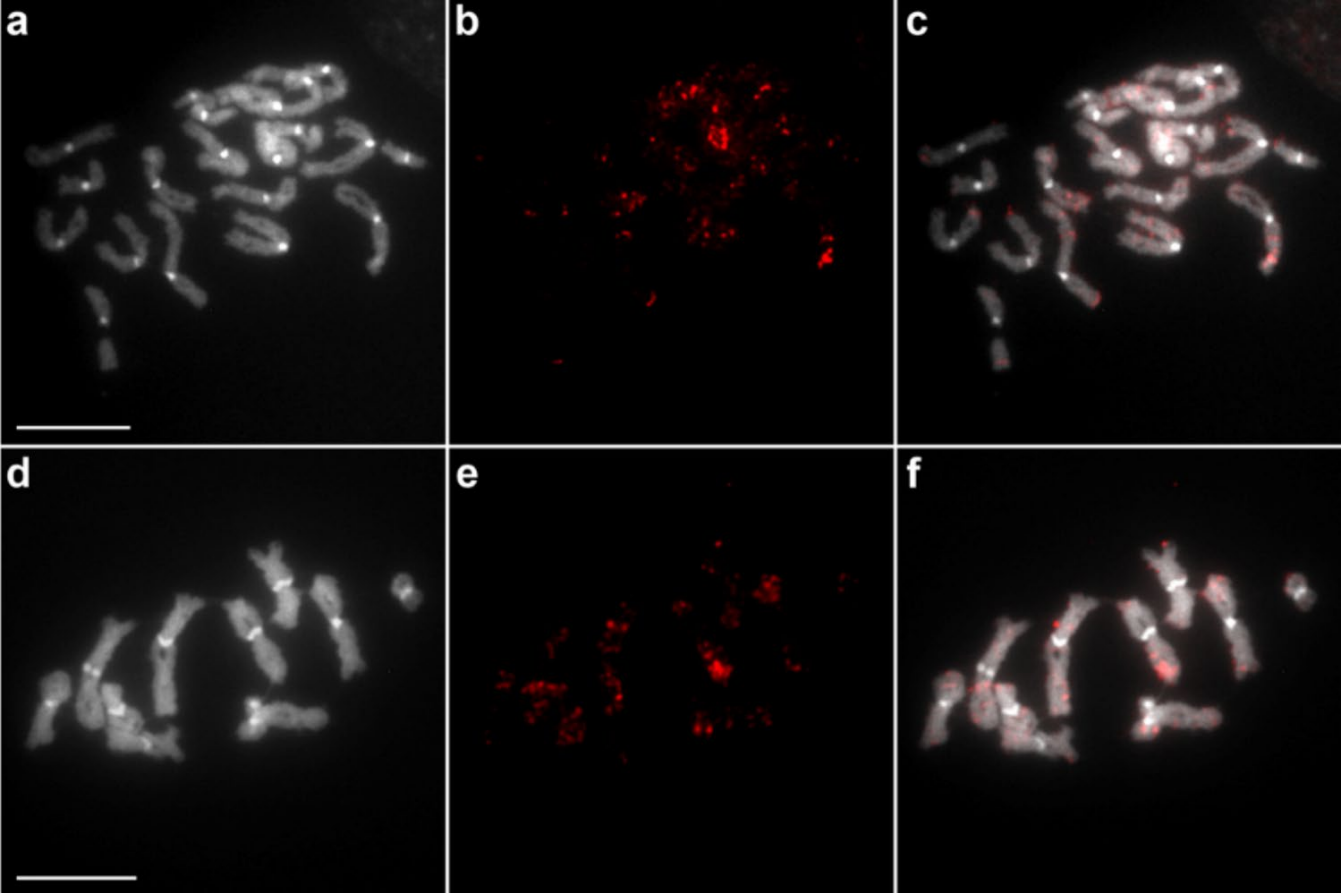
Chromosomal mapping of ClatSat02-1340 repeat on metaphase (**a**-**c**) and diplotene (**d**-**f**) chromosomes of *Caryophyllaeus laticeps*. (**a**, **d**) DAPI-stained chromosomes with visible centromeres, (**b**, **e**) probe signals for ClatSat02-1340, (**c**, **f**) merged images of DAPI and ClatSat02-1340 signals. The probe signals are enriched in subtelomeric and interstitial regions of the chromosomes. Scale bar = 10 μm

**Fig. 4:**
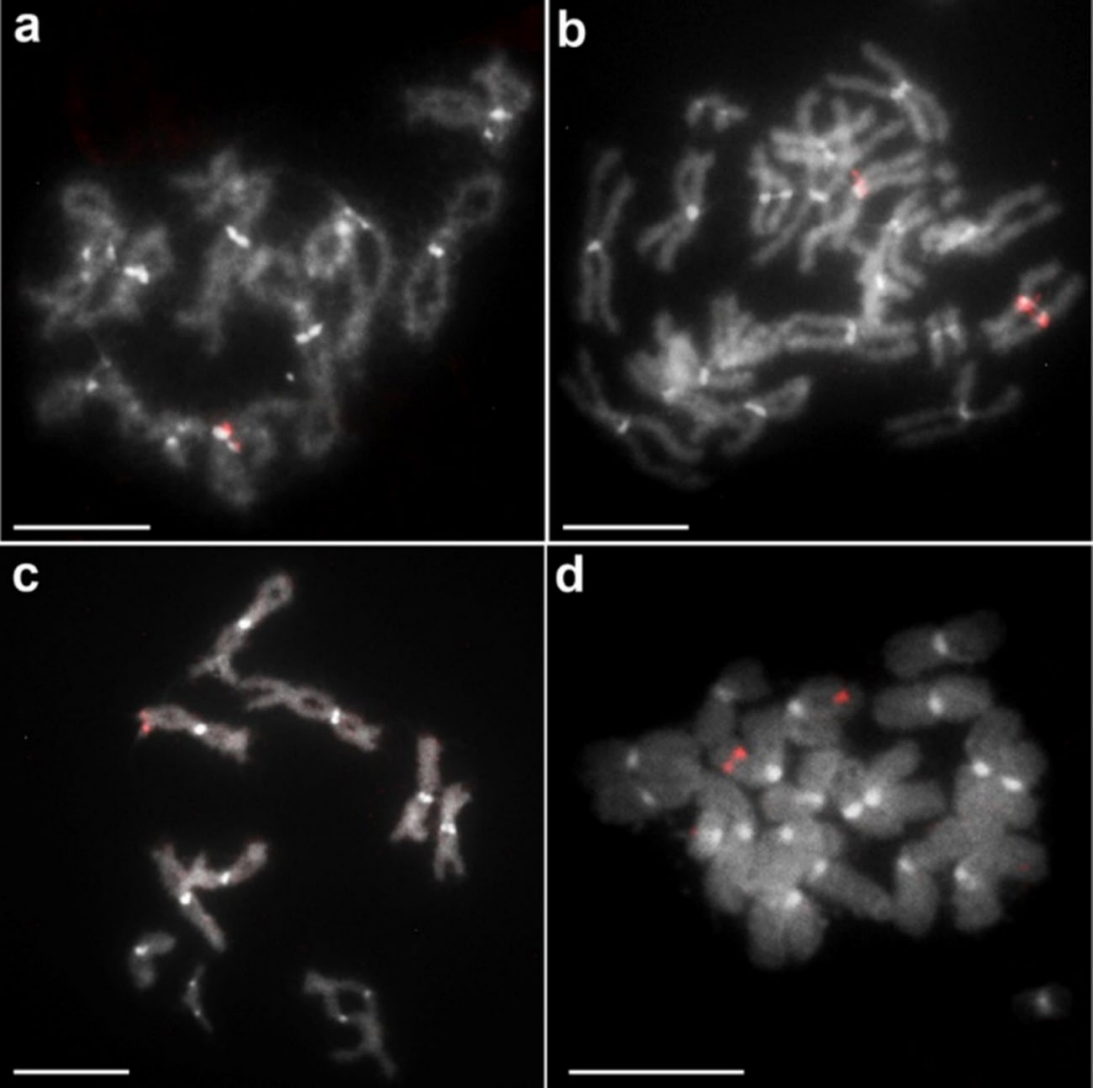
FISH mapping of U1 snDNA (**a**, **b**), ClatSat14-167 (**c**) and 5S rDNA (**d**) on chromosomes of *Caryophyllaeus laticeps*. (**a**) U1 snDNA signals on diplotene and (**b**) metaphase nucleus in pericentromeric region of chromosome pair No. 5, (**c**) ClatSat14-167 probe signals in the subtelomeric region of chromosome pair No. 4 during the diplotene stage (**d**) 5S rDNA signals in the subtelomeric region of chromosome pair No. 8. Chromosomes were counterstained with DAPI. Scale bar = 10 μm

**Fig. 5:**
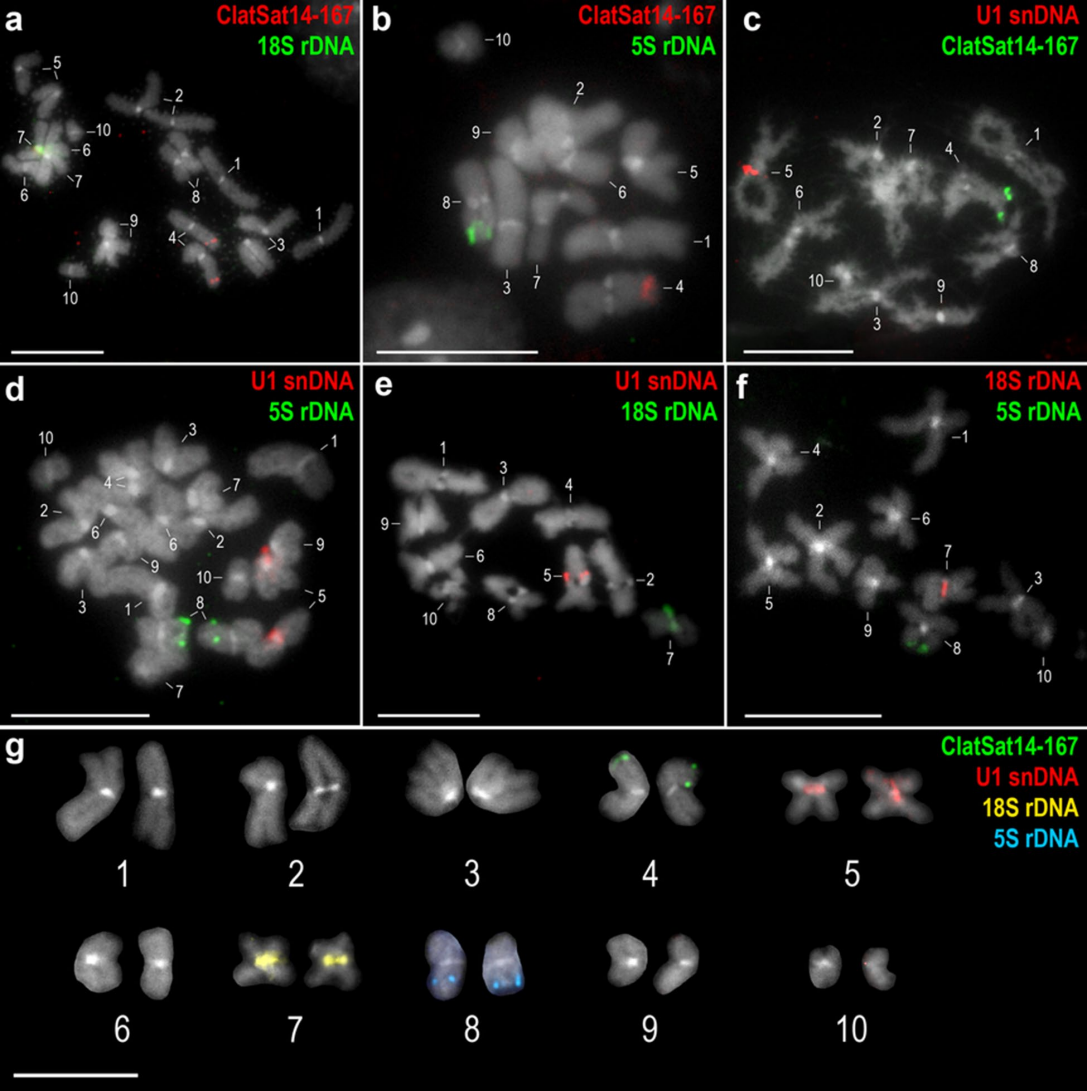
Two-color FISH combining ClatSat14-167, U1 snDNA, 5S rDNA, and 18S rDNA probes. (**a**) metaphase nucleus with hybridization signals for ClatSat14-167 (red) and 18S rDNA (green), (**b**) diplotene nucleus showing hybridization signals of ClatSat14-167 (red) and 5S rDNA (green), (**c**) diplotene chromosomes with U1 snDNA (red) and ClatSat14-167 (green), (**d**) metaphase nucleus with U1 snDNA (red) and 5S rDNA (green), (**e**) diplotene chromosomes with hybridization signals for U1 snDNA (red) and 18S rDNA (green), (**f**) metaphase II nucleus with hybridization signals for 18S rDNA (red) and 5S rDNA (green), (**g**) karyogram of *Caryophyllaeus laticeps* showing all chromosome markers. Chromosomes were counterstained with DAPI. Scale bar = 10 μm

The previously reported basic karyotypic characteristics of *C. laticeps* were confirmed, the diploid number of the analysed specimens was 2n = 20 and all chromosomes were confirmed to be metacentric. Of the 17 repetitive elements selected for mapping, eight (ClatTE01-4757, ClatSat02-1340, ClatTE02-4765, ClatSat05-467, ClatTE03-4582, ClatSat09-328, ClatSat13-761, ClatSat14-167) were successfully mapped to chromosomes (Figs. 1–4). FISH mapping showed a scattered distribution of most repeat families across all chromosomes. The putative LTR element ClatTE01-4757 (Fig. 1a,b) and the putative satellite ClatSat05-467 (Fig. 1c,d) showed abundant hybridisation signals on all chromosomes, without clear localisation patterns. The density of these repeats decreased with chromosome length, as observed in metaphase and diplotene nuclei (Fig. 1b,d). More subtle patterns were observed in certain dispersed repeats, such as the LTR elements ClatTE02-4765 (Fig. 2a,b) and ClatTE03-4582 (Fig. 2c,d) and the putative satellite ClatSat09-328 (Fig. 2e). ClatTE02-4765 and ClatTE03-4582 exhibited regions of enrichment located interstitially on the chromosome arms, with the exception of the centric and pericentromeric regions, which were most visible during metaphase and diplotene (Fig. 2a-d). ClatSat09-328 displayed stronger signals on the two largest chromosome pairs and four small metacentric pairs, which were mainly visible in diplotene (Fig. 2e). The putative satellite ClatSat02-1340 showed the most distinct distribution pattern, with enrichment in subtelomeric and interstitial regions clearly detectable in both metaphase and diplotene (Fig. 3). Three repetitive elements, ClatSat14-167, U1 snDNA and 5S rDNA, showed specific chromosomal localisation at single loci on the chromosomes of *C. laticeps* (Fig. 4). The hybridisation signals of U1 snDNA were detected in the pericentromeric position of a single metacentric chromosome pair No. 5 (Fig. 4a,b), most prominent in the diplotene (Fig. 4a) and metaphase (Fig. 4b) stages. The pericentromeric position was confirmed by DAPI staining, which highlighted the centromeres of all chromosomes. ClatSat14-167 showed clustered localisation at a single locus on metacentric chromosome pair No. 4 in the subtelomeric position, with signals most distinct at the diplotene stage (Fig. 4c). Finally, the 5S rDNA was detected in the subtelomeric region of a small chromosome pair No. 8 (Fig. 4d). To determine the exact position of each of these three repeats, two-colour FISH analysis was performed using combinations of ClatSat14-167, U1 snDNA, 5S rDNA and 18S rDNA probes (Fig. 5). A previous study on *C. laticeps* reported the localisation of the 18S rRNA genes in the pericentromeric region of chromosome pair No. 7 (Orosová et al. 2022). By comparing the chromosomal positions of the newly mapped repetitive elements with the known position of the 18S rDNA, the location of individual repeats was determined. Each repetitive family was mapped on distinct chromosome pairs (Fig. 5). Specifically, ClatSat14-167 was mapped to the subtelomeric region of chromosome pair No. 4 (Fig. 5a,b,c), 5S rDNA was localised to the subtelomeric region of chromosome pair No. 8 (Fig. 5b,d,f) and U1 snDNA hybridised to the pericentromeric region of chromosome pair No. 5 (Fig. 5c,d,e). The unambiguous localisation of each clustered repetitive family on the chromosomes enabled the construction of the primary karyogram of *C. laticeps* (Fig. 5g).

The chromosome slides prepared for FISH analysis, which were used to localise repetitive sequences, also showed the presence of chromosomal aberrations. Four different types of chromosomal aberrations were identified in the mitotic metaphases of *C. laticeps* (see supplementary material for detailed information).

## Discussion

### Repeatome analysis

Despite considerable attention being devoted to the genomic repeat content across various animal and plant species (Pamponét et al. 2019; Wang et al. 2022; do Nascimento Moreira et al. 2023; Mora et al. 2023; Voleníková et al. 2023; Zhang et al. 2023; Marková et al. 2025), the repeatome of tapeworms remains relatively underexplored. The available data come mainly from genomic studies, and only marginally focus on the repetitive content of several Cyclophyllidean species (Tsai et al. 2013; Zheng et al. 2013; Wang et al. 2016; Li et al. 2018; IHGC 2019; Maldonado et al. 2019; Nowak et al. 2019; Olson et al. 2020) and Diphyllobothriidean species (Bennett et al. 2014; IHGC 2019). In this study, repeatome analysis of short-read, low-coverage sequencing data from *C. laticeps* reveals a predominant presence of transposable elements (TEs), particularly retrotransposons. Specifically, we identified 98 clusters of LINE retrotransposons, 18 clusters of Ty3/Gypsy elements, and 32 clusters of Penelope elements. TEs are DNA sequences that are able to move within the genome by generating new copies of themselves. Their movement not only leads to the disruption of genes, but also plays a crucial role in regulatory changes, genome expansion and the generation of new chromosomal variants through mechanisms such as inversions. These elements serve as key drivers of gene evolution and the structural organisation of the genome. In addition, TEs can rapidly induce genomic changes (Flutre et al. 2011; Kapusta et al. 2013), particularly in response to environmental stressors, a concept first proposed by McClintock (1950). Activation of TEs by environmental stressors, which can trigger cellular stress and lead to DNA damage, can result in chromosomal rearrangements and genetic instability (Casacuberta and González 2013). Considering that *C. laticeps* and its fish host *A. brama* are constantly challenged by the presence of PCB chemicals in their environment, the involvement of TEs in adaptive responses to environmental challenges in the genome of *C. laticeps* is possible. TEs make up a considerable proportion of nuclear DNA in various taxa, up to 80% of the genome in plants, 3–20% in fungi and 3–52% in metazoans (Gregory et al. 2007; Wicker et al. 2007; Bennetzen and Wang 2014). The first published genomes of four tapeworm species showed a relatively low repeat content, ranging from 8 to 11% (Tsai et al. 2013). However, later studies using a combined sequencing strategy (with short Illumina reads and long PacBio reads) have shown that the percentage of repetitive sequences can exceed 20% (Li et al. 2018). More recently, the complete genome of *Hymenolepis microstoma* was published by Olson et al. (2020) and showed a high abundance of TEs, accounting for about 25% of the genome. Most TEs were highly dispersed, while some TEs were found at only a few sites or even a single site in the genome, consistent with our mapping of repeats, which we discuss below. Given these results, it is equally plausible that the high frequency of TEs in *C. laticeps* reflects an inherent genomic feature of tapeworm species rather than an adaptive response to environmental stressors. To better understand the underlying dynamics, comparative genomic analyses of different species and populations from environments with different levels of pollution in combination with advanced bioinformatics tools to identify and characterise repetitive elements are essential.

### Fluorescent mapping of the repetitive families onto chromosomes of *Caryophyllaeus laticeps*

The karyotype of *C. laticeps* is well known with a diploid chromosome number of 2n = 20 (n = 10 m) (Petkevičiūtė and Kuperman 1992; Bombarová and Špakulová 2015; Orosová et al. 2022). The chromosomes are relatively large, ranging from about 13 µm for the largest pair to 4 µm for the smallest (see Table 1 in Orosová et al. 2022), making them well suited for physical mapping of repetitive elements. Repetitive DNAs are often used as chromosomal markers because they are easy to isolate and can be mapped onto chromosomes using FISH. This powerful fluorescence mapping technique is a valuable technique for karyotyping, studying the fine structure of genomes and understanding their evolution. Repetitive DNAs are typically enriched in heterochromatic regions with low recombination rates on chromosomes, including centromeres, telomeric regions, sex chromosomes and supernumerary B chromosomes, although they can also be found in euchromatin (Biscotti et al. 2015; Hobza et al. 2017; Garrido-Ramos 2017; Marková et al. 2025). As already mentioned, knowledge regarding the distribution of repetitive DNA on the chromosomes of *C. laticeps*, but also in the entire group of Cestodes, is more than limited. In previous studies, only the location of 18S rDNA genes and GC- and AT-rich heterochromatin blocks were detected (Orosová et al. 2022). Fluorescence in situ hybridisation with an 18S rDNA probe revealed a single locus in the pericentric region of the small metacentric chromosome pair No. 7. Furthermore, the major ribosomal DNA was colocalised with GC-rich heterochromatin, suggesting a link between the major rDNA and GC-rich DNA. CMA_3_-positive sites were also detected in the telomeric regions of all chromosomes of *C. laticeps*, suggesting that not all GC-rich heterochromatin corresponds to nucleolar organiser regions (NOR). AT-rich heterochromatin was observed in the centromeric region of all chromosomes after DAPI staining (Bombarová and Špakulová 2015; Orosová et al. 2022). The present study represents a significant advancement to the expansion of this body of knowledge. The high sequence homology within certain repeat clusters enabled the development of fluorescently labelled probes and their mapping on the chromosome by FISH. Most of the mapped repeats showed a scattered distribution pattern (ClatTE01-4757, ClatSat02-1340, ClatTE02-4765, ClatSat05-467, ClatTE03-4582, ClatSat09-328), while three repetitive families exhibited a distinct chromosome-specific distribution in clusters (ClatSat13-761, ClatSat14-167, 5S rDNA). The repeat ClatSat13-761, characterised as U1 snDNA, showed a hybridisation signal at a single locus in the pericentromeric region of chromosome pair No. 5, ClatSat14-167 was located in the subtelomeric region of chromosome pair No. 4 and the 5S rDNA was assigned to the subtelomeric region of chromosome pair No. 8. Since some chromosome pairs in the species studied, apart from the largest and smallest pairs, have relatively small morphological and length differences, precise chromosome pairing and identification is relatively challenging. The development of these three new chromosome-specific markers helped in the accurate identification of three chromosome pairs, which represents a significant advance for the karyotyping of *C. laticeps*. Our study represents the first successful molecular characterisation and also chromosome mapping of 5S rRNA and U1 snRNA genes in a tapeworm genome. Both types of ribosomal genes (18S and 5S rDNA) are clustered at a single locus per haploid genome of *C. laticeps* and are located on different chromosome pairs. The number of rDNA loci is an important karyotypic feature. Although the number can vary, most species typically maintain a low number of rDNA loci (Sochorová et al. 2021). In terms of chromosomal positioning, numerous studies have documented both linked and unlinked arrangements of the 5S and 18S loci in different organisms (Srikulnath et al. 2009; García-Souto and Pasantes 2015; de Bustos et al. 2020; Orosová and Marková 2024), and these tandem arrays are more often found on separate chromosomes rather than being co-localised (Sochorová et al. 2018). Small nuclear RNAs (snRNAs) are highly conserved uridine-rich sequences that are components of the spliceosome, which consists of five snRNAs, U1, U2, U4, U5 and U6, that form its core structure (Watson et al. 2019). The main function of the spliceosome is to remove introns from the nascent RNA, a crucial step in mRNA maturation (West 2012). The snDNA belongs to a multigene family that is conserved in its sequence and has a variable number of repeats per genome. At the chromosomal level, U1 snRNA genes have been mapped in a limited number of species, including humans (Lund et al. 1983), mice (Lund and Nesbitt 1988), crustaceans (Barzotti et al. 2003), grasshoppers (Palacios-Gimenez et al. 2013; Anjos et al. 2015) and fish (Cabral-de-Mello et al. 2012), and have been evaluated as suitable markers for comparative cytogenetic studies. These studies have shown that the U1 snRNA genes are typically conserved with respect to their chromosomal location in related species and are often harboured on one or a few pairs of chromosomes. As hybridisation of 5S rDNA and U1 snRNA revealed a clustered organisation of these genes, both could serve as valuable markers to study chromosome evolution in tapeworms. With more data on additional species, this approach could become a powerful tool for answering evolutionary and taxonomic questions.

The application of next-generation sequencing in various taxa, including Acanthocephala, Cestoda and Trematoda, has greatly enhanced genomics research (Mauer et al. 2020; Kamenetzky et al. 2021; Solovyeva et al. 2021). However, molecular cytogenetic mapping remains limited, emphasising the need to map repetitive elements on chromosomes. Such studies are crucial for karyotyping, exploring chromosome and genome structure and performing evolutionary analyses (Muravenko et al. 2022; Samatadze et al. 2023; Mora et al. 2023; Marková et al. 2025). This study provides the first insights into the repeatome and chromosomal location of nine repetitive elements and identifies three as novel chromosomal markers, an important step towards the comprehensive characterisation of the tapeworm genome. In addition, the observed increase in chromosomal aberrations, likely linked to environmental pollution, underscores the need for further research on the genomic impact of environmental stressors.

## Supplementary Information

Below is the link to the electronic supplementary material.

Supplementary file1 (DOCX 521 KB)

## Data Availability

Sequence data supporting the results of this study have been deposited in the European Nucleotide Archive under the primary accession number PV288798 and PV396848.1. All sequenced data generated during the study are available from the corresponding authors.
