# Supplementary material for: Discovery of new chromosomal markers through repeatome analysis of Caryophyllaeus laticeps (Caryophyllidea)
Source: Parasitol Res. 2025 Jul 18;124(7):84. doi: 10.1007/s00436-025-08530-z (PMC12274263; doi:10.1007/s00436-025-08530-z)
Supplement: Supplementary file 1 — Supplementary file1 (DOCX 521 KB) [file 436_2025_8530_MOESM1_ESM.docx]

**Supplementary Materials** (Supplementary Figs S1 and Tables S1)

**Discovery of new chromosomal markers through repeatome analysis of *Caryophyllaeus laticeps* (Caryophyllidea)**

Anna, Marková ^ORCID^ **^0000-0002-0824-2026,^** ^1^, Martina, Orosová ^ORCID 0000-0001-8934-9126, 1*^

^1^Institute of Parasitology, Slovak Academy of Sciences, Hlinkova 3, 040 01 Košice, Slovakia;

^*^ Author for correspondence: Martina Orosová, E-mail: [orosm@saske.sk](mailto:orosm@saske.sk)

The Zemplínska Šírava reservoir, one of the most PCB-contaminated reservoirs in the world (Šalgovičová & Zmetáková 2006), served as the sampling site for this study as well as our earlier work (Orosová et al. 2022). In that study, we reported an increased frequency of chromosomal aberrations (CAs) in the *C. laticeps* population. In the present work, chromosomal slides prepared for FISH analysis, primarily used to localize repetitive sequences, again revealed CAs. Four distinct types of chromosomal aberrations were identified in the mitotic metaphases of *C. laticeps*: isochromatid gaps (ISCG), single chromatid breaks (SCB), single chromatid gaps (SCG), and double-strand breaks (DSB) (Supplementary Fig. S1a–d). These were present in preparations from four individuals. However, since CA detection was not the primary focus of this study, frequency data per 100 metaphases were not calculated.

Our recent research at this site (Orosová et al. 2022; 2023; Marková et al. 2024) supports a potential link between environmental stress and chromosomal instability. While B chromosomes were identified in two acanthocephalan species (*A. anguillae* and *A. lucii*), no structural aberrations were detected (Orosová et al. 2023; Marková et al. 2024). The same pattern was observed in *A. lucii* from the Ružín reservoir, another locality known for heavy metal contamination (Špakulová et al. 2002). The absence of visible CAs in acanthocephalans is likely due to the small size of their chromosomes, which limits cytogenetic resolution. Taken together, these findings reinforce the view that fish parasites may serve as sensitive indicators of environmental pollution and highlight a possible association between long-term PCB exposure and chromosomal damage in aquatic organisms.


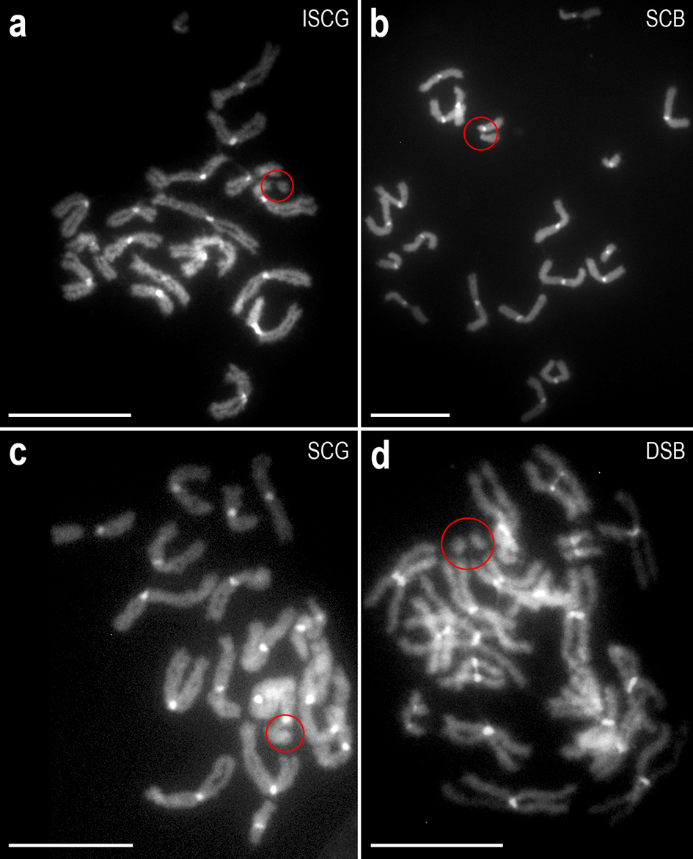


**Fig. S1** Detected chromosomal aberrations found in metaphase nuclei of *Caryophyllaeus laticeps*. (a) ISCG isochromatid gap, (b) SCB single chromatid break, (c) SCG single chromatid gap, (d) DSB double strand break. Chromosomes were counterstained with DAPI. Scale bar = 10 μm

| Cluster | TAREAN annotation | BLAST / GIRI results | Genome proportion  (%) | Monomer length  (bp) | Number of aligned reads | Chromosome distribution / number |
| --- | --- | --- | --- | --- | --- | --- |
| ClatSat01-136 | Putative satellite | not found | 2.60 | 136 | 12895 | failed to amplify |
| ClatTE01-4757 | Putative LTR element; Ty3_gypsy | not found / Gypsy-19_AmRa-I | **1.50** | **4757** | **7322** | strong dispersed signals over all chromosomes |
| ClatSat02-1340 | Putative satellite | not found | **1.30** | **1340** | **6516** | dispersed signals over all chromosomes, with enrichment in subtelomeric and interstitial regions |
| ClatSat03-331 | Putative satellite | not found | 1.10 | 331 | 5522 | amplified / not detected by FISH |
| ClatTE02-4765 | Putative LTR element; Ty3_gypsy | not found / Gypsy-5_TelSte-I | **0.99** | **4765** | **4964** | dispersed signals over all chromosomes, enrichments interstitially on chromosome arms |
| ClatSat04-299 | Putative satellite | not found | 0.90 | 299 | 4480 | failed to amplify |
| ClatSat05-467 | Putative satellite | not found | **0.76** | **467** | **3814** | strong dispersed signals over all chromosomes |
| ClatSat06-334 | Putative satellite | not found | 0.56 | 334 | 2809 | failed to amplify |
| ClatSat07-321 | Putative satellite | not found | 0.38 | 321 | 1925 | failed to amplify |
| ClatTE03-4582 | Putative LTR element; Ty3_gypsy | not found / Gypsy-5_FaGi-I | **0.31** | **4582** | **1562** | dispersed signals over all chromosomes, enrichments interstitially on chromosome arms |
| ClatSat08-4433 | Putative satellite | not found / Gypsy-5_TelSte-I | 0.27 | 4433 | 1367 | failed to amplify |
| ClatSat09-328 | Putative satellite | not found | **0.23** | **328** | **1159** | dispersed signals over all chromosomes stronger signals on the two largest chromosome pairs and four small metacentric pairs |
| ClatSat10-533 | Putative satellite | not found | 0.09 | 533 | 451 | failed to amplify |
| ClatSat11-445 | Putative satellite | not found | 0.07 | 445 | 344 | amplified / not detected by FISH |
| ClatSat12-1926 | Putative satellite | not found | 0.064 | 1926 | 318 | amplified / not detected by FISH |
| ClatSat13-761 | Putative satellite | U1 small nuclear RNA | **0.053** | **761** | **264** | single locus in the centromeric region / No. 5 |
| ClatSat14-167 | Putative satellite | not found | **0.052** | **167** | **258** | single locus in the subtelomeric region / No. 4 |
| 5S |  | 5S rDNA |  |  |  | single locus in the subtelomeric region / No. 8 |
| 18S |  | 18S rDNA |  |  |  | single locus in the centromeric region / No. 7 |

**Table S1** Repeats selected for mapping on chromosomes of *Caryophyllaeus laticeps*

*** **Bold** numbers indicate repeats that were successfully detected with FISH method

**References**

Marková A, Orosová M, Marec F, Barčák D, Oros M (2024) Karyological study of *Acanthocephalus lucii* (Echinorhynchida): the occurrence of B chromosomes in populations from PCB-polluted waters. Diversity 16:140. <https://doi.org/10.3390/d16030140>.

Orosová M, Marková A, Marec F, Barčák D, Brázová T, Oros (2022) New cytogenetic data on *Caryophyllaeus laticeps* and *Paracaryophyllaeus gotoi*, parasites of evolutionary interest. Parasitology 149:1094-1105. https://doi.org/10.1017/S0031182022000622.

Orosová M, Marková A, Zrzavá M, Marec F, Oros M (2023) Chromosome analysis and the occurrence of B chromosomes in fish parasite *Acanthocephalus anguillae* (Palaeacanthocephala: Echinorhynchida). Parasite 30:44. https://doi.org/10.1051/parasite/2023045.

Šalgovičová D, Zmetáková Z (2006) Polychlorinated biphenyls is in muscle tissue of freshwater fish in east Slovakia. J Food Nutr Res 45:171-178.

Špakulová M, Kráľová-Hromadová I, Dudiňák V, Reddy PV (2002) Karyotype of *Acanthocephalus lucii*: the first record of supernumerary chromosomes in thorny-headed worms. Parasitol Res 8:778–780. https://doi.org/10.1007/s00436-002-0639-y.
